# Response to the Letter to the Editor by Eberhard et al.

**DOI:** 10.1186/s13071-017-2125-5

**Published:** 2017-05-16

**Authors:** Christian Bottomley, Valerie Isham, Sarai Vivas-Martínez, Annette C. Kuesel, Simon K. Attah, Nicholas O. Opoku, Sara Lustigman, Martin Walker, Maria-Gloria Basáñez

**Affiliations:** 1 MRC Tropical Epidemiology Group, London School of Hygiene and Tropical Medicine, Keppel Street, London, WC1E 7HT UK; 2 Department of Statistical Science, University College London, Gower Street, London, WC1E 6BT UK; 3 Cátedra de Salud Pública. Facultad de Medicina (Escuela Luis Razetti), Universidad Central de Venezuela, Caracas, Venezuela; 4 UNICEF/UNDP/World Bank/WHO, Special Programme for Research and Training in Tropical Diseases, World Health Organization, Geneva, Switzerland; 5 Department of Microbiology, University of Ghana Medical School, Accra, Ghana; 6 University of Health and Allied Sciences Research Centre (UHASRC) Hohoe, Volta Region, Ghana; 7 Laboratory of Molecular Parasitology, Lindsley F. Kimball Research Institute, New York Blood Center, 310 E 67th St, New York, NY10065 USA; 8 London Centre for Neglected Tropical Disease Research, Department of Infectious Disease Epidemiology, School of Public Health, Faculty of Medicine (St Mary’s campus), Norfolk Place, London, W2 1PG UK; 9 Department of Pathobiology and Population Sciences and London Centre for Neglected Tropical Disease Research, Royal Veterinary College, Hawkshead Lane, Hatfield, Hertfordshire AL9 7TA UK

## Abstract

In a Letter to the Editor, Eberhard et al. question the validity of our model of skin snip sensitivity and argue against the use of skin snips to evaluate onchocerciasis elimination by mass drug administration. Here we discuss their arguments and compare model predictions with observed data to assess the validity of our model.

## Letter to the Editor

In our recent paper in *Parasites & Vectors* [1], we presented a model of the number of microfilariae (mf) per skin snip, which we used to predict the sensitivity of skin snips as a test for detecting patent *Onchocerca volvulus* infection when a treatment programme, based on mass drug administration (MDA) with ivermectin, is close to achieving elimination. We concluded that our model supports the recommendation of the African Programme for Onchocerciasis Control (APOC) to conduct skin snip surveys 3–5 years post-MDA, and that sensitivity could be improved by taking four skin snips rather than two. Based on the findings from our model, we argued that skin snips are useful, when used together with entomological and serological data, for evaluating programmes of MDA.

In a Letter to the Editor, Eberhard and colleagues [2] suggest that our model of skin snip sensitivity is misspecified, and argue that skin snips should not be used for evaluating onchocerciasis elimination by mass drug administration because they have poor sensitivity and individuals are often reluctant to be skin-snipped.

**Fig. 1:**
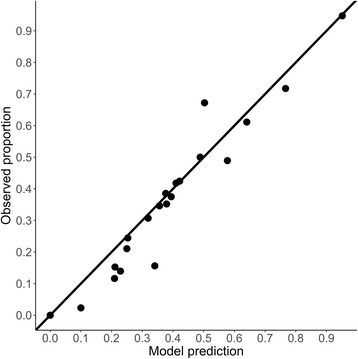
Proportion of iliac skin snips with zero microfilariae versus model predictions for study communities in Guatemala, Venezuela, Cameroon and Ghana

Eberhard et al. begin their critique by suggesting our model is misspecified. In particular, they question our assumption that the number of mf per skin snip follows a negative binomial distribution (conditional on adult female worm burden) and suggest that a zero-inflated distribution might be more appropriate. As we discussed in our paper, there are good theoretical reasons for choosing this model, and it fits the data well. Specifically, it does not underestimate the proportion of skin snips with zero mf (Fig. 1), as the authors suggest it might. We believe a zero-inflated model is therefore unnecessary.

The authors then present two arguments against the use of skin snips for evaluating programmes of MDA. Their first argument is that skin snip sensitivity is known to be low. But they present an estimate of sensitivity (20%) without reference to either time after treatment or number of skin snips. By itself, this figure is uninformative since, as we have shown, sensitivity ranges from zero to *c.*100%, depending on the number of skin snips taken and when they are taken after treatment. For two skin snips, our model predicts low sensitivity (≤ 31%) one year after treatment, which is consistent with data presented by Thiele et al. [3].

Their second argument is that skin snips are not useful because people are sometimes reluctant to be skin-snipped; they cite as evidence the low rates of participation in the Mali/Senegal study [4, 5] (*c*.70% participated in the first survey conducted after the last round of MDA and *c*.50% in the fourth skin snip survey 3–4 years later). We acknowledged this limitation in our paper when we said that, “communities are increasingly reluctant to participate in skin snipping, so a compromise must be found between what is feasible and the ideal” and that “strategies and surveillance should be implemented using tests that are less invasive than the skin snip method.” Nonetheless, we believe skin snip data from the Mali/Senegal study are useful since they were used successfully to model the likelihood of elimination and recrudescence [6], and the model predictions are now supported by Ov16 serological data from Senegal indicating that elimination may not have been sustained in the River Gambia focus [7].

Eberhard et al. conclude that “country program managers must be made aware of the *extreme* lack of usefulness of skin snips in assessing the elimination of *Onchocerca volvulus*”. However, the recommendation of the World Health Organization (WHO) that skin snip microscopy should not be used to demonstrate interruption of transmission [8] is only a “conditional recommendation, low certainty of evidence”. Equally, program managers should be made aware of the limitations of Ov16 serology, particularly of the current rapid diagnostic tests. The WHO recommendation to use Ov16 serology in < 10-year olds to confirm transmission interruption is a “strong recommendation”, but also based on “low certainty of evidence”, and it may need to be adapted in the future. For example, the WHO guidelines provide a single threshold of Ov16 seropositivity (<0.1%); however, recent modelling suggests that it may be more appropriate to use thresholds that depend on pre-MDA endemicity [9, 10]. Clearly, more research is needed to achieve ‘high certainty of evidence-based recommendations’. Our study contributes to this endeavour.
